# Policy recommendations for sustainable livestock farming in South Korea: review

**DOI:** 10.5713/ab.25.0307

**Published:** 2025-09-30

**Authors:** Inkuk Yoon, Jong Hyun Jung, Sang-Hyon Oh, Sung Woo Kim

**Affiliations:** 1 Department of Animal Science, North Carolina State University, Raleigh, NC, USA; 2 Gyeongnam Provincial Government, Changwon, Korea; 3 Jung P&C Institute, Yongin, Korea; 4 Division of Animal Science, Gyeongsang National University, Jinju, Korea

**Keywords:** Digital Transformation, Economic Incentives, Manure Management, Public-private Governance, South Korea, Sustainable Livestock Farming

## Abstract

This study aimed to investigate the transition towards sustainable livestock farming, emphasizing the role of policy instruments, challenges in implementation, and future directions. There are many ways to help move towards sustainable livestock farming. The primary methods in major Organization for Economic Co-operation and Development (OECD) countries are regulation, economic incentives, and supportive measures. The most important tools are regulation and financial support. This study looked at the effectiveness of these tools, the role of government in supporting sustainable livestock, and ways to improve these policies. It also discussed using smart technology for sustainable farming, introducing a certification system for sustainable livestock products, increasing public interest and willingness to pay for sustainable products, training future experts, and creating partnerships between the public and private sectors. The study concluded that effective policy implementation requires a combination of regulation and support. Necessary regulations should be applied with enough time and agreement from society, even if the livestock industry opposes. Support policies are currently scattered and not well-connected, so they need to be comprehensively linked to sustainable livestock farming. It is important to have a consistent policy system that sets measurable goals, provides budget support, and evaluates performance step by step to achieve the goal of sustainable livestock farming in South Korea.

## INTRODUCTION

Various policy instruments are available to promote the transition to sustainable livestock farming [1]. Among these, major OECD member countries predominantly utilize regulations, economic incentives, and promotional measures as primary policy tools [2,3]. The most critical policy instruments in this context are likely to be regulations and financial support. Regulations may include imposing specific requirements or permitting conditions for certain economic activities to achieve objectives, levying taxes or penalties for environmental burdens, and establishing environmental cross-compliance obligations. Cross-compliance obligations refer to additional conditions that must be fulfilled when implementing specific measures. Regulatory policy instruments are primarily applied in areas where negative externalities occur and are considered to be effective in addressing these issues.

By imposing taxes or obligations to address the negative externalities resulting from agricultural and livestock management, policymakers ensure that farmers and livestock producers perceive these costs as part of their operational expenses. In response, farmers engage in a series of agricultural management practices aimed at reducing costs to secure economic viability for their farm incomes [4]. This approach leads to the internalization of external factors within the value chain of agricultural management [5].

On the other hand, economic incentives and promotional measures include financial supports such as direct subsidies, operating fund support, credit loans or interest subsidies, tax or utility fee reductions, and direct payment systems, as well as support for technology and research and development [6].

One of the most representative economic incentive policies implemented in OECD member countries, which does not distort market prices but is based on the multifunctional role of agriculture, is the direct payment system [6–8]. For instance, livestock managers receive agricultural subsidies under the condition that they produce public benefits or values. Direct payment systems extend beyond simple farm income support or price support subsidies, aiming to achieve public value based on the multifunctional role of agriculture and livestock [5]. This policy is widely utilized in many countries. It is generally accepted as it involves supplying public services or goods to a certain socially required level for the public good, and economic incentives tend to have higher acceptance among agricultural and livestock stakeholders compared to regulations [8,9]. Therefore, this paper proposes policies for sustainable livestock farming in South Korea.

## THE EFFECTIVENESS OF POLICY MEASURES FOR SUSTAINABLE LIVESTOCK FARMING

There is considerable debate regarding the policy effectiveness and validity of regulatory and economic incentive measures [10]. Regulatory policies can reduce social costs through preemptive measures that establish certain standards or conditions. However, these policies may encounter strong resistance from stakeholders during implementation. On the other hand, economic incentive policies are generally well-received by stakeholders due to their alignment with their preferences. Nevertheless, these policies face limitations in ensuring their effectiveness and often face criticism regarding the appropriateness of budgetary expenditures from external sources [11].

To achieve the policy effects of sustainable livestock farming through these two policy instruments, it is essential to first establish consistent policy principles during the design of regulatory and economic incentive policies [9].

If the government fails to obtain societal consensus and stakeholder cooperation during the implementation of the policy, maintaining the consistency of the policy becomes challenging, potentially leading to revisions, deviations, or prolonged implementation delays. Therefore, it is crucial to establish firm policy principles and conduct thorough research and cross-analysis of external factors and diverse stakeholder interests when determining appropriate measures through the coexistence of regulatory and economic support policies [12].

Clear establishment of policy principles is essential, and the decision-making process should involve not only the primary stakeholders, such as farmers and livestock producers, but also the government, consumer organizations, private sectors, social organizations, and relevant businesses. Engaging all these stakeholders in setting a common direction and reaching a consensus is crucial. Only through such a process can the procedural and logical legitimacy of various policy measures aimed at promoting the transition to sustainable livestock farming be recognized, thereby increasing policy acceptance [13].

When implementing financial incentive support policies, the following three principles must be adhered to in order to achieve higher policy effectiveness and enhance budget execution effectiveness. First, financial incentives should be provided when additional costs and special efforts are incurred to create public benefits. Second, there must be a potential social demand for the public benefits that the policy supported by the incentives is expected to generate. Third, there should be potential expected outcomes where additional or enhanced public benefits can be achieved as a result of the incentive support [9,14,15].

## JUSTIFICATION FOR GOVERNMENT INTERVENTION IN SUSTAINABLE LIVESTOCK FARMING

There is likely to be little resistance from stakeholders, such as farmers and livestock producers, regarding economic incentive policies due to the fact that these policies involve some level of budgetary support for farm incomes [16]. However, it is more challenging to build consensus on regulatory policies, particularly concerning the scope, intensity, and timing of regulations. There tends to be a fundamental reluctance towards government intervention in these cases.

The most commonly applied principle in the implementation of regulatory policies is the “polluter pays” principle [17]. When it is possible to directly identify the sources of environmental pollution, such as the discharge of livestock wastewater or pollutants, it is straightforward to apply this principle to hold the polluters accountable for the damage caused. However, there remains controversy over whether this principle can be applied to cases where environmental damage is difficult to specify or occurs in small-scale or minimal quantities within the scope of private property rights. This issue ultimately relates to the legitimacy of government intervention or infringement on private property rights.

Particularly, the potential for social constraints on land ownership has been a central issue in the implementation of agricultural policies. Since land is an essential factor for agricultural activities, especially South Korea’s geography is quite mountainous and not abundant in livestock-capable land, this concern is even more pronounced [17].

In the debate between public and private interests, the principle of sustainable development has recently been applied. This principle addresses whether individual agricultural or livestock management activities can proceed independently of environmental or social considerations. Specifically, it refers to the process of harmonizing economic growth with environmental and social values, ensuring fairness and equity among current and future generations. Social values imply providing equal opportunities to all members of society and ensuring intergenerational equity, which considers the welfare of future generations [18].

Under this concept, the principle of sustainable development provides significant insights into enforcing regulations and imposing costs associated with livestock management activities to promote multifunctionality and sustainability in agriculture and livestock management [17,19]. Even if environmental pollution resulting from livestock management activities remains confined to an individual’s land or facility, the actions causing pollution diminish opportunities and values for future generations, thus necessitating regulation [17]. Adhering to the general principles of sustainability, which advocate for environmentally friendly conservation of agricultural and natural resources, maintenance of sustainable ecosystems, and equitable use across generations and social strata, can establish the legitimacy of regulatory measures and restrictions on private rights [20,21].

## ENHANCING THE EFFECTIVENESS OF POLICY MEASURED FOR SUSTAINABLE LIVESTOCK FARMING

Currently, the primary policy measures employed in South Korea for promoting environmentally friendly and sustainable livestock farming include economic incentives such as financial support, environmental regulations, certification support, as well as research, development, and technological assistance [22]. In South Korea, various financial support measures are implemented to promote sustainable livestock farming. These include subsidies for building smart livestock facilities [23], supporting livestock manure treatment facilities [24], resource utilization and energy facilities for manure [25], and agricultural material costs [26]. Additionally, support through the public interest subsidy system for environment-friendly, safe livestock products, regional subsidies, and management stability funds are also in place [22].

Regulations related to livestock farming primarily consist of environmental regulations aimed at reducing soil, water, and air pollution according to relevant laws. Additionally, there are limitations on livestock management practices and conditions to reduce environmental burdens imposed alongside various financial support measures. However, in South Korea, there are currently no regulations regarding greenhouse gas emissions, nor are there established measurement methods, standards, or institutional mechanisms for assessing reductions in emissions [27].

Although there are regulations related to livestock product certification, most of these rely on voluntary compliance, and the certification standards are somewhat ambiguous. There is a lack of quantifiable and clear criteria for these standards [28].

To promote sustainable livestock farming, it is essential to provide sufficient economic incentives and financial support for livestock farmers to transition from conventional to sustainable practices. A stable transition is possible when farmers have the expectation that their income will either be maintained or increased from an economic perspective.

Furthermore, the current certification standards need to be clarified, and stronger penalties and corresponding disadvantages should be imposed for environmental pollution, regulatory violations along with exceeding regulatory limits to ensure the effectiveness of the policies.

Since 2022, the state government of California in the US has been implementing legislation to prevent intensive confinement farming, also known as factory farming, by ensuring adequate space for pigs, cattle, and chickens [29]. Similarly, the EU has been gradually banning the use of small cages for poultry farming since 1999, and many European countries are in the process of enacting national legislation to prohibit small cage systems individually [30]. These developments indicate that governments, facing limitations of existing certification systems based on voluntary participation and self-regulation, are attempting to drive structural changes in farming practices through legislative amendments. However, it is important to note that such stringent regulations inevitably lead to conflicts with the livestock industry [21].

As evidenced by international examples, the effectiveness and consistency of policy implementation are best achieved when regulation and support are pursued in tandem. In this regard, despite potential resistance from the livestock industry, it is essential to implement necessary regulations alongside support measures, following sufficient transition periods and social consensus.

Furthermore, the current livestock-related support measures are fragmented by sector and livestock type, with insufficient integration between them. Therefore, it is necessary to develop comprehensive support schemes that align with sustainable livestock practices, similar to the Environmental Quality Incentives Program (EQIP) in the US [31,32]. Livestock farmers should be supported by the administration to develop their own sustainability implementation plans, which, after government review, can be supported through comprehensive financial assistance, thereby enhancing policy effectiveness. In other words, under the goal of transitioning to sustainable livestock farming, detailed implementation plans should be established at the farm level, and tailored support measures should be adopted.

It is also essential to establish a coherent policy support system with quantifiable goals and detailed objectives for each implementation stage, including budget support, regulations, and incentives. This system needs to incorporate phased support, performance evaluation, and feedback mechanisms to ensure consistency and effectiveness.

## IMPROVING THE LIVESTOCK ENVIRONMENT FOR SUSTAINABLE LIVESTOCK INDUSTRY

### Background on livestock environmental improvement

The primary challenge currently confronting the livestock industry is the management of animal manure and odor issues. These challenges represent significant obstacles to both the sustainability of livestock farming and the expansion of the industry. Particularly with regard to greenhouse gas emissions, there is a vigorous international movement towards reduction [27]. Nationally, failure to reduce carbon emissions could lead to the imposition of a carbon tax and the emergence of new trade barriers, which are expected to have substantial impacts on the domestic economy. Therefore, addressing manure management through resource recovery and energy conversion while simultaneously resolving odor problems is essential for the continued viability of the livestock industry.

According to the Food and Agriculture Organization (FAO), as of 2019, the total greenhouse gas emissions from the livestock sector are estimated at 3.44 billion tons, with enteric fermentation contributing 3 billion tons (87.2%) and manure management accounting for 440 million tons (12.8%). In contrast, data from the Greenhouse Gas Inventory and Research Center of Korea for 2020 indicates that in South Korea, emissions from manure management (approximately 4.94 million tons) surpass those from enteric fermentation (approximately 4.47 million tons). This discrepancy is attributed to the higher proportion of pig and poultry farming compared to beef and dairy cattle. Therefore, the effective reduction of greenhouse gases in the Korean livestock sector hinges on proper manure management, resource recovery and energy conversion. This paper aims to propose solutions for improving the livestock environment with a focus on reducing manure and greenhouse gas emissions as critical components for sustainable livestock farming.

### Establishing an integrated manure management system

According to a 2022 survey conducted by the Ministry of Agriculture, Food and Rural Affairs (MAFRA), the total annual livestock manure production in South Korea amounted to 50,732 thousand tons. Among this, pigs accounted for the largest share at 37.9% (19,210 thousand tons), followed by beef and dairy cattle at 34.2% (17,349 thousand tons), poultry at 18.8% (9,555 thousand tons), and dairy cows at 9.1% (4,618 thousand tons) (Table 1). Of the total manure produced, 52.1% (26,426 thousand tons) is managed on-farm by the producers, while the remaining 47.9% (24,306 thousand tons) is handled through outsourcing to manure treatment facilities [33].

By livestock type, cattle and dairy farms that also engage in crop farming and own farmland have high on-farm treatment rates, with 81.7% for beef cattle and 85% for dairy cattle. Poultry farms also show a high on-farm treatment rate of 82%, as manure is often processed into compost. In contrast, pig and duck farms have lower on-farm treatment rates at 66.2% and 68.9%, respectively. This is attributed to factors such as farm aging and stricter environmental regulations, leading to a higher reliance on outsourced manure management.

The majority of processed manure is utilized as compost (75.3%) and liquid fertilizer (11.7%) for land application, while the remaining 13% undergoes treatment through purification processes. As the number of livestock continues to rise, the volume of livestock manure generated also increases. However, the available agricultural land for spreading manure, which is predominantly utilized for compost and liquid fertilizer, is decreasing. This discrepancy highlights the urgent need for diverse resource management strategies, including the energy recovery of livestock manure, to address the growing challenge. According to predictions based on the current trend of decreasing agricultural land and regional livestock manure generation, it has been projected that by 2030, 73 counties will face a shortage of agricultural land available for the application of compost and liquid fertilizers [34].

Due to the varying scales of livestock farming and manure generation, as well as differences in agricultural land and nutrient requirements across counties, it is essential to establish an integrated livestock manure management system to ensure efficient manure treatment.

Based on the results of the livestock manure survey, it is necessary to analyze trends such as regional livestock scales, manure generation, and the area of farmland required for compost and liquid fertilizers. Subsequently, comprehensive measures for improving the livestock environment should be developed. These measures should include regional manure management strategies, solutions for reducing livestock odors, and plans for greenhouse gas emission reduction, all of which should be integrated into a unified management system [35].

In the current situation, the handling of livestock manure that exceeds self-processing capacities must be expanded through alternative methods of resource recovery or energy utilization. Despite its public benefits, communal manure resource recovery projects have faced severe opposition from local residents due to odor complaints, leading to significant delays in these projects. Since 2007, 34 project plans have been abandoned [36]. Additionally, as greenhouse gas emissions from the livestock sector continue to rise, there is an increasing demand for new reduction methods, including the utilization of livestock manure for renewable energy. It is now essential to introduce various manure treatment methods beyond traditional compost and liquid fertilizer, such as direct treatment, biochar, bioplastics, biogas, and solid fuels [37].

If livestock manure energy projects are implemented in conjunction with the Ministry of Environment’s eco-friendly energy town development projects, it could enhance both the effectiveness of the projects and community acceptance. Results from the pilot project in Hongcheon indicate that producing bioenergy from livestock manure, converting it to city gas, and supplying it to local residents, along with creating revenue through local village projects utilizing electricity or waste heat, have proven effective [38]. Therefore, it is necessary to expand these findings nationwide based on more detailed result analyses. Additionally, similar to the bio-plant case in Hongseong, where methane gas generated from livestock manure processing is used for electricity production, such methods should be adapted and expanded according to regional characteristics [38].

Currently, it is necessary to consolidate livestock manure treatment facilities from individual farms into a regional or multi-county integrated livestock manure management system. By doing so, it will be possible to balance the quantity of livestock manure generated and treated at the county or regional level and establish a more efficient treatment system.

### Establishing a livestock odor management system

Livestock odor issues depend on improvements in feed, maintaining a clean livestock environment, and the proper treatment of livestock manure [39,40].

Firstly, reducing odor through feed improvement is a key strategy [39]. Research is underway on various feed improvement methods, such as low-methane feed, protein-reduced feed, nitrogen emission reducers, microbial additives, and fermented carbohydrate supplements. Among these, low-methane feed has proven effective and is being distributed to farmers [36].

However, the effectiveness of these feed additives often relies on unilateral results from manufacturers. Therefore, it is crucial for organizations such as the National Institute of Animal Science, regional Agricultural Research & Extension Service, and regional Agricultural Technology Centers, along with livestock organizations, to collaborate in conducting empirical tests and pilot projects. This will help objectively verify the effectiveness and analyze the economic feasibility of these additives, thereby expanding their adoption.

Secondly, managing a clean livestock environment and applying appropriate feeding techniques are crucial. Maintaining an optimal stocking density is a priority in livestock environment management. When the number of animals exceeds the capacity that a facility can handle and manage, odor issues are exacerbated, and animal welfare is compromised. Installing odor-reducing equipment can also be a solution for managing livestock environments [29,31]. Currently, available technologies and devices include liquid fertilizer recycling systems, bio-curtains, biofilters, scrubbers, plasma-based odor reduction devices, and cartridge exchange-based odor reduction systems. Various technologies are being applied in the field.

Despite significant investments in facilities and expenses to reduce livestock odor, it remains a challenging issue at the farm level. Since livestock farms differ in terms of scale, management practices, and facility structure, there is a need for tailored odor-reduction technologies and implementation models [41]. This requires collaborative consulting involving relevant institutions, organizations, and experts to support customized solutions.

Thirdly, proper management of livestock manure treatment is essential. It is necessary to analyze the comprehensive conditions, including the specific circumstances of livestock farms, surrounding farmland, and the utilization of energy facilities, to apply the optimal manure treatment method [19,21,42]. As previously mentioned, treating manure at the individual farm level has limitations in terms of efficiency and cost-effectiveness. Therefore, establishing an integrated manure treatment system at a regional or multi-county level is crucial.

## ACCELERATING THE ADAPTATION OF SUSTAINABLE DIGITAL LIVESTOCK FARMING THROUGH SMART TECHNOLOGIES

The integration of ICT technologies, including big data, artificial intelligence, and blockchain, into the livestock sector is transforming the industry. This integration enables the development of remote and automated livestock management systems that maintain and manage optimal animal husbandry environments. Additionally, it strengthens the safety of livestock infrastructure and conserves energy through the combination of hardware and software systems. As a result, digital livestock farming is becoming increasingly established [43,44].

The application of digital livestock farming encompasses a range of technologies, including various sensors and monitoring devices for measuring environmental conditions both inside and outside the barn, as well as digital production management facilities such as milking machines, automated feed management systems, automatic feeders, appropriate biometric selectors, pregnancy detection devices, and biometric activation sensors. Additionally, it includes advanced technologies like robotic milking machines, water management systems, electronic tags such as collars or implants, and individual animal management systems. The scope of digital livestock farming continues to expand with the integration of these specialized management systems and the development of comprehensive farm management and decision-making systems that provide necessary solutions.

The digital livestock market is experiencing a global expansion trend, and in South Korea, various startups and IT companies are developing sector-specific solutions. However, the domestic digital livestock sector remains in its early stages, predominantly relying on products developed abroad [44]. While the swine and poultry sectors, which have long been industrialized and scaled, are rapidly advancing in mechanization, automation, and digitalization, the dairy and beef cattle sectors still heavily depend on manual labor and remain in the initial stages of development.

As the number of livestock farms rapidly declines and the farming population ages, it is essential to leverage digital livestock technologies to address labor shortages. Utilizing digital technology is crucial for managing various aspects of sustainable livestock farming, including animal husbandry, environmental management, and greenhouse gas reduction. Employing these technologies will enhance the competitiveness of livestock management by providing comprehensive management functions necessary for effective and sustainable farm operations.

Currently, digital livestock farming in South Korea primarily focuses on monitoring technologies through sensors and automation [43]. However, the practical application of data-driven digital technologies such as big data, artificial intelligence, and blockchain remains limited [44]. The ultimate goal of digital livestock farming is to develop platforms incorporating autonomous management systems through big data analytics and AI, as well as integrated management traceability systems using blockchain. Advanced countries have moved beyond merely inventing monitoring solutions to develop application software and leverage big data and AI. As a latecomer, South Korea needs to prioritize bold R&D investments and focus on product development through field demonstrations.

To advance digital livestock farming, it is crucial to expand pilot and demonstration projects across all possible areas in collaboration with domestic digital livestock companies. Solutions developed by digital technology firms must undergo field trials to ensure practical applicability [43,44]. Government support policies that mitigate development risks are also necessary. Utilizing various testbeds and accumulated experience from demonstration projects will expedite the real-world application of digital livestock technologies.

If producer organizations such as Agricultural Cooperatives, livestock associations by species, central government, and local governments collaborate to establish a roadmap for digital livestock farming and share resources for its implementation, it will enable a more efficient realization of digital livestock practices [44].

In South Korea, numerous digital livestock companies and startups, such as AIDKOREA (livestock health care solutions company), Intflow (livestock AI specialist company), uLikeKorea (digital livestock company), and Hankook IoT Cort. (integrated ICT convergence system company for livestock), are developing new products, conducting field trials, and even pursuing international exports.

By collaborating with these companies to replace existing imported products with domestic alternatives and expanding into new areas of digital livestock farming, a new market that extends the scope of the livestock sector could be established.

## INTRODUCTION OF SUSTAINABLE LIVESTOCK PUBLIC-INTEREST DIRECT PAYMENT

### Current status of public-interest direct payment in South Korea

In South Korea, the public-interest direct payment is implemented and designed to provide financial support to farmers for creating public benefits such as maintaining rural areas, environmental conservation, and food safety through agricultural activities. This system is divided into basic and optional types (Table 2). The basic type includes small farmer subsidies and area-based subsidies, while the optional type includes subsidies for eco-friendly farming, environment-friendly safe livestock products, landscape conservation, and strategic crops [45]. In the livestock sector, the only subsidy available is the environment-friendly safe livestock products subsidy, which is provided to farms producing organic-certified livestock products with Hazard Analysis Critical Control Point (HACCP) certification.

The environment-friendly safe livestock products subsidy system is designed to compensate for the decrease in initial income and production cost differences for eco-friendly livestock farms. This system aims to promote the expansion of eco-friendly farming and establish a sustainable foundation for environmental conservation in livestock production [45].

Implemented in 2009, this system has faced several practical limitations and issues, including the effectiveness of payment rates, restrictions on organic maintenance subsidies, excessive costs for certification, and difficulties in obtaining organic certification. Due to these complex factors, the number of farms receiving eco-friendly safe livestock products subsidies has stagnated. This stagnation may indicate that, from an economic perspective, the system has not provided sufficient incentives for livestock farmers.

### Introduction of a sustainable livestock public subsidy system

To transition and expand from conventional livestock farming to sustainable livestock farming promptly, it is necessary to implement a “Sustainable Livestock Public Subsidy System” similar to the public welfare subsidies or incentive support systems used in advanced countries [46]. The current public subsidy system provides only minimal income support to farmers certified in organic livestock farming, lacking sufficient economic incentives for general livestock farmers to transition. To achieve sustainable livestock farming, it is essential to provide corresponding economic incentives for realizing public values such as food security, environmental conservation, food safety, and climate crisis response.

In advanced countries, eco-friendly livestock subsidy programs are supported to achieve various policy goals, including addressing environmental issues such as soil, water, air pollution, and odors, ensuring food safety, improving animal welfare, and managing land and landscapes [35,47]. Additional incentive support systems for various public welfare livestock activities, such as environmentally friendly feed production, pasture management, livestock circular agriculture, and ensuring transparency and reliability in livestock management through digital technologies, are needed.

Carbon-neutral implementation programs for low-carbon farming and livestock activities should be included in the public subsidy system to encourage greenhouse gas reduction. Adopting a scoring system similar to the U.S. EQIP to provide incentives for various sustainable livestock activities offers valuable policy insights [31].

Establishing a sustainable livestock public subsidy system requires government funding, and thus, a social consensus on supporting livestock farmers must be achieved before implementation [46,48]. Specific quantification of the public value brought by sustainable livestock farming is necessary, as well as an analysis of the potential impacts on livestock scale expansion resulting from the public subsidies.

Additionally, activities aimed at reducing the negative externalities of conventional livestock farming could also be eligible for public subsidies. For example, activities such as livestock manure energy recovery, odor reduction, greenhouse gas mitigation, digital technology-based safety management, and animal welfare implementation contribute to realizing public value by mitigating existing externalities [46–48].

However, to effectively reduce negative externalities, it is essential to conduct specific verification and assessment of the environmental and ecological impacts, as well as the potential losses that livestock farmers may experience. This detailed evaluation is necessary to establish practical public subsidies and determine appropriate support levels.

The introduction of a sustainable livestock public subsidy system requires thorough research on preconditions and policy effects, supported by field demonstrations through pilot projects [46,49].

The introduction of a sustainable livestock public subsidy system requires thorough research on preconditions and policy effects, supported by field demonstrations through pilot projects. Furthermore, to establish sustainable livestock farming on-site, the current public subsidy support levels and budget may serve as effective incentives and income protection under normal circumstances. However, without risk management measures to address large domestic animal diseases or drastic price drops due to external factors, sustainable livestock farming may revert to traditional practices in times of crisis. Therefore, the development of risk mitigation tools, such as self-help insurance or guarantee systems, is also necessary. The U.S. provides various forms of price support for agricultural and livestock products, including dairy revenue protection programs, support for damages from retaliatory trade, and price decline insurance programs.

In addition to the burdens on farms participating in sustainable livestock farming and government budget support, it is necessary to consider implementing price risk guarantee programs to support farm income in the event of rapid declines in sustainable livestock product prices.

### Decentralization of the authority for implementing sustainable livestock public subsidy systems

In South Korea, the public subsidy system is managed centrally by the MAFRA, which controls all authorities and resources according to a unified national standard. Local governments are responsible only for the execution according to laws and guidelines [43]. Given that agricultural and livestock conditions vary by region and that the scale and management methods of livestock farming differ, it is necessary to decentralize the authority for not only the sustainable livestock public subsidy system but all public subsidy systems to local governments. This would allow local authorities to promote agricultural policies with autonomy and independence under their own responsibility. It is crucial to develop customized policies for detailed implementation plans, address urgent issues, determine transition methods, and establish agreements with livestock farmers on the roadmap for transition.

If the central government provides comprehensive guidelines and resources and delegates authority to local governments, these local authorities can adjust subsidies or implement infrastructure support projects based on different livestock species, farming methods, and carbon reduction technologies. For example, if transitioning to sustainable livestock practices is urgent in the swine industry, there are significant negative externalities, additional incentives or budget support specific to this sector could be provided. Similarly, if certain R&D technologies are needed, extra funding for these endeavors could be allocated.

Similarly, the support levels or budget criteria for the public subsidy system could be adjusted according to the characteristics of each local government. Rather than having a uniform support level or criteria across all regions, differential approaches based on local conditions might be more effective in achieving policy goals.

If policies tailored to the conditions of each region are developed and implemented, a nationwide transition to sustainable livestock farming can be achieved more quickly. Especially if specialized pilot models for sustainable livestock farming are established in different regions, they can be disseminated to other regions, promoting broader adoption. Additionally, distributing risks across all regions can enhance the efficient allocation of budgets [50].

## INTRODUCTION OF THE “SUSTAINABLE LIVESTOCK CERTIFICATION SYSTEM” FOR SUSTAINABLE LIVESTOCK FARMING

### Current status of the livestock certification system in South Korea

**Figure 1:**
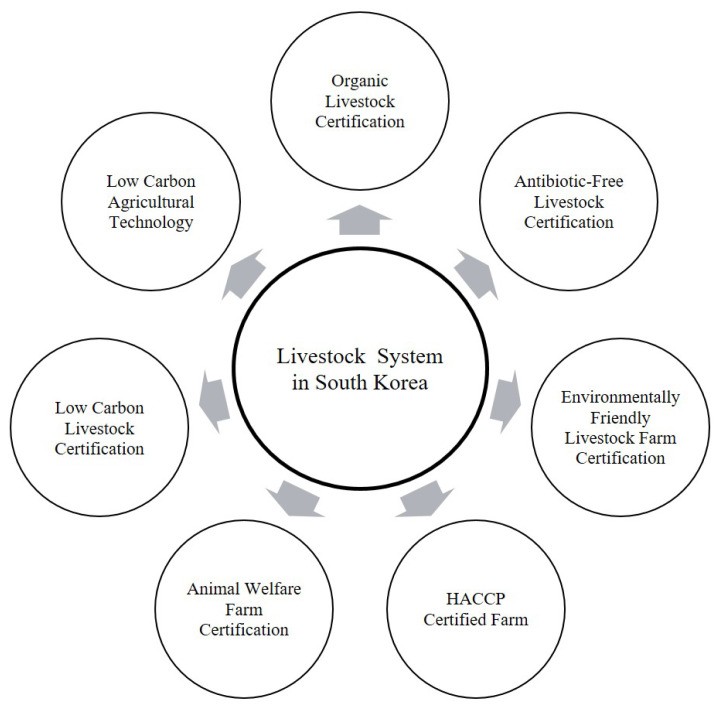
The livestock certification system implemented in South Korea. HACCP, Hazard Analysis Critical Control Point.

Currently, South Korea has several certification systems related to hygiene, safety, and environmental aspects in livestock farming, including organic livestock products, antibiotic-free livestock products, environmentally friendly livestock farms, clean livestock farms, HACCP-certified farms, and animal welfare farms (Figure 1). Starting in 2012, South Korea is implementing a pilot project for a low-carbon livestock product certification system aimed at supporting carbon reduction in the livestock sector, specifically targeting Hanwoo cattle farms.

Under the “Framework Act on Carbon Neutrality and Green Growth for Climate Crisis”, low-carbon agricultural and livestock products are defined as those produced using low-carbon agricultural technologies specified by the law, resulting in greenhouse gas emissions lower than the standard emission levels for the respective products.

Until now, the livestock sector has not implemented low-carbon livestock product certification due to the lack of clearly defined detailed standards for low-carbon livestock technologies [51].

In this pilot project, the criteria for low-carbon livestock product certification require farms that have already received environmental or hygiene and safety certifications to apply at least one of the carbon reduction technologies recognized by the MAFRA. To qualify for certification, farms must reduce their greenhouse gas emissions by at least 10% below the average emission levels for their livestock type [52]. Carbon reduction technologies that comply with government standards include improvements in feed management, such as the use of low-methane feed, reduction of rearing periods, production of biochar from manure, appropriate composting techniques, and the adoption of high-efficiency energy equipment, all in accordance with regulations established by the Intergovernmental Panel on Climate Change (IPCC).

### Direction for introducing a sustainable livestock product certification system

The currently piloted low-carbon livestock product certification system is in its initial stages [35,52]. A thorough analysis of the pilot project’s outcomes is necessary for its refinement and improvement. Additionally, there is a need to extend the pilot program to other livestock sectors, such as swine, poultry, and dairy farming, at an early stage. The current pilot project alone has significant limitations in promoting the transition to sustainable livestock farming. Given that it is conducted in separate areas—such as livestock type, rearing stages, manure and energy—the effectiveness of the policy is highly restricted. Ultimately, to transition to sustainable livestock farming, an integrated certification system is required that covers all stages of production, including feed production, rearing methods, manure management and energy utilization, and the production of safe livestock products. The existing certification system focuses primarily on livestock farms or production stages. Consumers will likely prefer products that are certified as sustainable across all stages of production, processing, and distribution.

However, the current certification system only establishes standards and implements certification for certain aspects such as production methods, processing methods, livestock farm environments, and animal welfare [46]. As a result, consumers lack reliable information on other aspects of the product. This limitation affects the willingness to pay additional costs and the expansion of social demand. For example, while consumers may purchase organic livestock products, they do not receive additional information on whether the product fully meets animal welfare standards during rearing, whether the treatment and utilization of livestock manure adhere to reasonable criteria, or whether digital technologies are used to address disease, hygiene, and safety issues during the rearing process. Therefore, reliable information on all processes and detailed aspects of livestock products can be provided using blockchain technology, making the implementation of a sustainable livestock certification system feasible.

Existing certification systems have been established through numerous trials and errors and are currently in place in various fields, having achieved a certain level of consumer awareness and trust. Therefore, it is necessary to maintain these existing systems as they are.

The newly introduced sustainable livestock certification system can encompass existing certification systems while adding standards for areas not covered, such as circular agriculture, resource recovery and energy utilization of livestock manure, and the implementation of digital livestock technologies. Similar to the phased certification system for environmentally friendly agricultural products in its early stages (initially involving low-pesticide, no-pesticide, and organic products) the sustainable livestock certification system should also be divided into 3 to 4 stages to establish an implementation framework.

### Introduction of sustainable livestock farm certification system

The implementation of a sustainable livestock product certification system must prioritize the sustainability of the farming stage. Only livestock products produced on farms certified as sustainable livestock farms can receive the sustainable livestock product certification. Currently, in South Korea, certification systems for environmentally friendly livestock practices include certifications for eco-friendly farms, clean farms, HACCP-certified farms, animal welfare farms, and low-carbon livestock product farms [53].

To be designated as an eco-friendly livestock farm, a farm must secure a substantial area of land and separate processing facilities, which involves significant initial investment costs. In response, the MAFRA has recently relaxed some of the criteria and introduced the “Clean Livestock Farm” system. Clean livestock farms are those that comply with density regulations, properly manage manure, and harmonize with the surrounding environment, according to standards set by the MAFRA. After designation, additional investments and certifications are progressively conducted, with a phased approach toward transitioning to eco-friendly livestock farms [33,34]. The government’s phased introduction of the system, including a transitional model, is considered a measure to enhance the system’s activation by considering the practical conditions of livestock farmers. However, even when compared to advanced countries, the standards for eco-friendly livestock farms set by the MAFRA have limitations in achieving truly eco-friendly operations. Particularly in terms of sustainable livestock farming standards, there is still a significant gap between the goals. Thus, there is a need for the introduction of a more robust system with enhanced criteria that can be implemented on a broader scale, tailored to the specific conditions of South Korea [34].

Through the past cases of the eco-friendly agricultural certification system implemented since 2001, the initial phase faced challenges in transitioning from conventional agriculture. To address this, the system included low-pesticide certification within its scope, allowing for a phased transition through low-pesticide certification, no-pesticide certification, and organic certification. However, about 60% of certified farms remained at the low-pesticide stage, and following the discontinuation of new low-pesticide certifications in 2010 and the complete abolition of the low-pesticide certification system in 2016, many farms reverted to conventional agriculture [54]. Some transitioned to the less stringent GAP certification, failing to reach the final goal of organic or no-pesticide certification. A thorough analysis of why the eco-friendly certification system had limitations in market demand and why farm certifications did not increase is crucial [15]. This analysis will serve as a foundation for the implementation of eco-friendly livestock farms and, ultimately, sustainable livestock farming.

If livestock producers do not comply with the certification requirements and there is no willingness to pay for such products in the market, the initiative is likely to face difficulties. Similarly, if various livestock product certification systems fail to generate new demand in the market and if the costs and investments are not matched by a willingness to pay, these certification systems will not become established and will ultimately fail to be implemented in the livestock sector.

If livestock product certification systems are based on criteria and implementation at the farm level or small regional units, there will be limitations to expanding certified farms and products. It is now necessary to implement a sustainable livestock farm certification system at a broader regional level. Additionally, for constraints such as separate farm-level processing facilities or feed self-sufficiency, it is important to provide policy and institutional support by establishing these facilities at a regional level or recognizing supply within the region, allowing livestock farmers to focus solely on rearing and production [50].

## WILLINGNESS TO PAY AND EXPANSION OF SOCIAL DEMAND FOR SUSTAINABLE LIVESTOCK PRODUCTS

The primary requirement for achieving sustainable livestock farming is ensuring the economic viability of livestock farmers. To transition from conventional to sustainable livestock farming, it is essential that current income levels are maintained or improved. Securing economic viability for livestock farmers ultimately depends on the corresponding willingness to pay and consumption in the market. To support farmers in bearing the additional costs associated with sustainable practices, it is crucial to create social demand that can uphold prices through increased consumer willingness to pay.

According to Sung [55], the willingness to pay for sustainable livestock farming and consumer willingness to pay for sustainable livestock products is notably high. When estimating the amount consumers are willing to pay for different types of livestock, the amounts were found to be between 3.7 and 4.4 trillion won (2.85 and 3.38 billion US dollars approximately) for pork, 3.4 to 3.9 trillion won (2.62 to 3.00 billion US dollars approximately) for beef, and 3.6 to 4.2 trillion won (2.77 to 3.23 billion US dollars approximately) for chicken. This indicates a significant consumer demand for sustainable livestock products. However, it is crucial to note that the willingness to pay does not always align with actual consumer spending. Thus, the challenge lies in effectively translating consumer willingness to pay into actual consumption.

As previously discussed, in the United States, consumer willingness to pay for livestock products produced through sustainable management is clear in the market. The costs associated with sustainable production are reflected in the consumer prices of these products [49,56].

Ultimately, creating demand in the consumer market for sustainable livestock products requires addressing consumer awareness issues, communication with consumer organizations, conducting nationwide promotions, and fostering ongoing awareness changes in local communities. Additionally, establishing networks with distribution channels such as organic specialty stores, online shopping platforms, and large retailers is crucial. Targeted marketing strategies for consumers of certified sustainable livestock products should also be continuously pursued. This includes integrating sustainable practices into school meal programs to educate future consumers about sustainable diets. The success of domestic dairy products, like the Im-sil cheese, in carving out niche markets amidst the dominance of imported dairy products, illustrates the potential for domestic livestock markets. Expanding the market for sustainable livestock products through the development of processed goods, rather than merely selling meat products, is likely to be more effective. Exploring opportunities in the processed meat market, such as developing new products and substituting imports, could also be a viable strategy.

In the United States, the key to securing and expanding a stable consumer base for sustainable livestock products has been effective collaboration with distribution partners. By supplying products through organic specialty retailers and stores and targeting specific consumer segments through marketing strategies, the US has successfully expanded its market. Collaborating with franchise operators, food service companies, large retailers, and organic specialty stores to develop and offer products like organic livestock, low-carbon certified meat, and other sustainable livestock products, as well as creating new items, could be highly effective in securing demand [22]. Designing the entire value chain (from production and processing to distribution) based on sustainability principles can help create a new “blue ocean” in the sustainable livestock consumption market.

## NURTURING FUTURE KEY WORKFORCE FOR SUSTAINABLE LIVESTOCK FARMING

The number of livestock farms is declining each year, and the aging of the farming population is accelerating. According to the ‘2022 Agriculture, Forestry, and Fisheries Survey’ published by Korea Statistical Information Service (KOSIS, Statistics Korea), the number of livestock farms decreased by 4.7% (1,023,000 households) from the previous year, with the number of elderly farmers (aged 65 and above) reaching about half of the total, marking an era of an aging farming population. However, the number of successor or new startup entrants is decreasing. Particularly, due to worsening domestic and international environmental conditions and declining economic viability, the number of small-scale livestock farms closing down is increasing, and the influx of new young people into the livestock industry is sharply declining. For example, in the past year alone, around 2,000 small-scale farms with fewer than 50 cattle closed. In the dairy sector, more than 300 farms have closed over the past two years. Specifically, in the dairy sector, the proportion of farmers aged 60 and above is increasing annually, while the proportion of dairy farmers under 30 has dropped to 10%. In the pig farming sector, only 16% of farmers were under 50 years old as of last year [57].

According to a 2022 survey conducted by the Korean Pork Future Research Institute, approximately 60% of pig farms, which are among the most scaled-up sectors in livestock farming, reported a lack of successor farmers. In the poultry sector, the number of small-scale farms has significantly decreased, and there are only about 2,200 full-time poultry farms nationwide that raise more than 10,000 birds each [58].

In the U. S., there is also a significant shortage of new livestock farmers, leading to special support and benefits for young and new entrants into the industry. Similarly, the United Kingdom, once a leading country in pig farming, faced a severe crisis due to a sharp decline in successor farmers in the pig industry. This shortage of successors was one of the factors that led to the halving of the pig farming industry’s scale.

In the European Union (EU), young farmers under the age of 40 account for only 11% of the total, with a rapid decline in the number of farmers and increasing aging of the farming population. To support the influx of young people into agriculture, the EU operates various programs, including agricultural startup grants, farm income support, and various training programs. Notably, since 2015, the EU has introduced a young farmers’ subsidy scheme. The upper limit of the total subsidies has been increased from 2% to 3%, and up to an additional 25% of subsidies can be allocated to young farmers on top of existing farm subsidies.

To ensure the survival of the livestock industry, the influx of new and young personnel is crucial. The government needs to implement tax benefits, regulatory adjustments, policy considerations, and diverse development programs to foster successor livestock farmers and attract new entrants [1,2,48]. The ‘Youth Settlement Support Program’, which has been in effect since 2018, should be restructured and the support amounts should be adjusted to reflect current realities.

Furthermore, the ‘Act on the Promotion and Support of Successor and/or Young Workforce’, which has been in place since 2021, outlines a plan for fostering young farmers at the regional level. However, due to inadequate stable financial support from the central government, these programs have shown limited effectiveness. Therefore, it is essential to provide separate financial support from the government and develop comprehensive support measures through coordination among relevant departments. Additionally, segmented support policies should be integrated and provided comprehensively across different times and areas [59].

In order to facilitate the influx of new personnel into the livestock industry, it is essential to have supporting infrastructure such as farmland and livestock facilities. Therefore, there is a need to expand farmland support through the Farmland Bank and to establish a system for transferring and inheriting existing livestock facilities to new personnel. Furthermore, it is necessary to broaden the current selection-based support system for young farmers to provide equal opportunities for all eligible young farmers, similar to the approach taken by the EU, and to address the barriers in the support programs for young farmers.

Now, as the livestock industry transitions to digital agriculture and smart facilities, it is essential to establish institutional support measures to increase the participation of female livestock farmers [60]. The traditional male-dominated livestock environment has become more accessible to women due to advancements in digital and automation technology. Therefore, it is necessary to develop and operate programs that provide special opportunities and support for women in order to nurture elite future talent.

## ESTABLISHING A PUBLIC-PRIVATE GOVERNANCE FOR SUSTAINABLE LIVESTOCK FARMING

Sustainable livestock farming cannot achieve its intended goals through government-led regulations or the enactment of relevant laws alone. It is essential for on-site livestock farmers to recognize the necessity of participating voluntarily in the transition towards sustainable practices.

Currently, livestock-related consultative groups are primarily operated separately by species, such as Hanwoo, pig, poultry, dairy, and beef cattle. There is also a lack of coordination and collaboration with general agriculture sectors or organic farming organizations [60,61]. Producer organizations like the National Agricultural Cooperative Federation (NACF) or National Livestock Cooperatives Federation (NLCF) are centralized at the national level but operate separately at the regional level. Additionally, organizations related to sustainable livestock farming, including other agricultural sectors, consumer groups, relevant institutions, environmental groups, and animal welfare organizations, each operate their own consultative groups.

Initially, it is crucial to organize consultative groups for sustainable livestock farming by species to build internal consensus and create a roadmap. Subsequently, there should be an expansion of networks through collaboration with relevant institutions and organizations in agriculture, consumer sectors, distribution, environment, and animal welfare.

As illustrated by the case of the United States, transitioning to sustainable livestock farming necessitates the establishment of a public-private governance framework involving all relevant organizations. This includes livestock-related associations, sustainable agriculture groups, agricultural and livestock cooperatives, animal welfare non-profit organizations, environmental organizations, consumer groups, community-based organizations, distribution companies, local universities, research institutions, central government, and local governments [60].

It is essential to establish and operate an integrated offline consultative group for sustainable livestock farming. Additionally, creating an online platform for sustainable livestock farming that allows all relevant stakeholders to participate can facilitate discussions on common interests, seek consensus, and lead legislative and policy improvements.

## CONCLUSION

As seen in various cases, regulation and support must be combined to ensure consistency and effectiveness in policy implementation. From this perspective, even if there is opposition from the livestock industry, necessary regulations should be implemented in parallel with sufficient implementation periods and social consensus. Furthermore, since current livestock-related support policies are scattered across different sectors and types of livestock, and there is a lack of connectivity between these policies, it is necessary to establish comprehensive support policies linked to sustainable livestock farming. Policy effectiveness can be enhanced by enabling livestock farmers to prepare their own sustainable livestock implementation plans with administrative support and having the government review these plans to provide comprehensive financial support. In other words, it is necessary to establish a consistent policy support system that sets quantifiable goals and detailed objectives for each implementation stage, along with budget support, regulations, and incentives, and conducts step-by-step support, performance evaluation, and feedback under the goal of transitioning to sustainable livestock farming in South Korea.

## Figures and Tables

**Table 1:** Annual production of livestock manure by the Ministry of Agriculture, Food and Rural Affairs (MAFRA) in South Korea in 2022

Livestock type	Amount (thousand ton)	Percentage
Pig	19,210	37.9
Beef cattle	17,349	34.2
Poultry	9,555	9.1
Diary cow	4,618	18.8
Total	50,732	100.0

Data from MAFRA [62].

**Table 2:** Public direct payment system in South Korea

Type	Division
Optional type	Environment-friendly safe livestock direct payment Direct payment for Eco-friendliness Direct payment for Rice paddy utilization Direct payment for Rural landscape conservation
Basic type	Direct payment for small-scale farms Area-based direct payment

Data from MAFRA [63].
